# Shortwave infrared diffuse optical wearable probe for quantification of water and lipid content in emulsion phantoms using deep learning

**DOI:** 10.1117/1.JBO.28.9.094808

**Published:** 2023-06-12

**Authors:** Samuel S. Spink, Anahita Pilvar, Lina Lin Wei, Jodee Frias, Kylee Anders, Sabrina T. Franco, Olivia Claire Rose, Megan Freeman, Grace Bag, Huiru Huang, Darren Roblyer

**Affiliations:** a Boston University, Department of Biomedical Engineering, Boston, Massachusetts, United States; b Boston University, Department of Electrical and Computer Engineering, Boston, Massachusetts, United States

**Keywords:** diffuse optics, diffuse optical imaging, wearable, shortwave infrared, shortwave infrared, water, lipid, deep learning

## Abstract

**Significance:**

The shortwave infrared (SWIR, ∼900 to 2000 nm) holds promise for label-free measurements of water and lipid content in thick tissue, owed to the chromophore-specific absorption features and low scattering in this range. *In vivo* water and lipid estimations have potential applications including the monitoring of hydration, volume status, edema, body composition, weight loss, and cancer. To the best of our knowledge, there are currently no point-of-care or wearable devices available that exploit the SWIR wavelength range, limiting clinical and at-home translation of this technology.

**Aim:**

To design and fabricate a diffuse optical wearable SWIR probe for water and lipid quantification in tissue.

**Approach:**

Simulations were first performed to confirm the theoretical advantage of SWIR wavelengths over near infrared (NIR). The probe was then fabricated, consisting of light emitting diodes at three wavelengths (980, 1200, 1300 nm) and four source-detector (S-D) separations (7, 10, 13, 16 mm). *In vitro* validation was then performed on emulsion phantoms containing varying concentrations of water, lipid, and deuterium oxide ($D_{2}O$). A deep neural network was developed as the inverse model for quantity estimation.

**Results:**

Simulations indicated that SWIR wavelengths could reduce theoretical water and lipid extraction errors from ∼6% to ∼1% when compared to NIR wavelengths. The SWIR probe had good signal-to-noise ratio (>32  dB up to 10 mm S-D) and low drift (<1.1% up to 10 mm S-D). Quantification error in emulsion phantoms was 2.1±1.1% for water and −1.2±1.5% for lipid. Water estimation during a $D_{2}O$ dilution experiment had an error of 3.1±3.7%.

**Conclusions:**

This diffuse optical SWIR probe was able to quantify water and lipid contents *in vitro* with good accuracy, opening the door to human investigations.

## 1 Introduction

The near infrared (NIR, ∼700 to 1000 nm) wavelength band has dominated diffuse optical imaging and spectroscopy in tissue for decades. While often utilized to quantify hemoglobin, NIR technologies have also been used to measure water and lipid *in vivo*, *ex vivo*, and *in vitro*, typically using wavelengths in the 900 to 1000 nm range.^1^^–^^7^ The accurate quantification of tissue water and lipid is of interest, as this capability could enable a wide range of clinical, point-of-care, and consumer applications including volume status monitoring for end-stage kidney disease and heart failure patients, tissue hydration monitoring for athletes, body composition assessment during weight loss, cancer treatment monitoring, and others.^8^ With respect to clinical applications, kidney disease patients undergoing dialysis treatment experience fluid accumulation and require fluid removal, making frequent water volume assessment a desirable capability. Heart failure patients experience similar fluid accumulation, and water and lipid content have been shown to have prognostic value for monitoring breast cancer treatment response during neoadjuvant chemotherapy.^1^^,^^2^ With respect to consumer applications, serious and casual athletes alike are continuously searching for methods of performance enhancement, with hydration optimization and continuous body composition analysis offering obvious benefits in this field.

The shortwave infrared (SWIR, ∼900 to 2000 nm) wavelength band is currently being explored as an alternative to the NIR due in part to the recent improvements in the availability and performance characteristics of SWIR-active detectors. Advantages of the SWIR wavelength band include relatively low tissue scattering,^9^ which has been utilized for deep tissue fluorescence microscopy with applications including the imaging of cancer biomarkers, vascular disorders, liver disease, and bone structure.^10^^–^^13^ Water and lipid also gain dominance as absorbing chromophores in the SWIR, which suggests that their estimation *in vivo* with a wearable device would benefit from exploiting this optical window. It should be noted that the SWIR window of particular interest in this work (∼900 to 1300 nm) is sometimes referred to as the second NIR window, and that additional SWIR windows (or NIR windows, depending on the arbitrary naming convention being used) at longer wavelengths also exist, generally located between sequential water absorption peaks. However, references to the “NIR” region in this study refer to the traditional first NIR window. Prior work employing SWIR spectroscopy for water and lipid quantification includes the use of frequency-domain (FD) measurements for absolute absorption and scattering coefficient estimation, as well as hyperspectral continuous wave (CW) measurements employing spectral constraints.^7^^,^^14^ Our prior work has demonstrated water and lipid quantification using a technique called hyperspectral SWIR spatial FD imaging. While these prior investigations show promise, clinical and at-home translation in this space remains limited in part due to the complexity of current measurement techniques, which may require bulky spectrometers, custom electronics, and expensive scientific grade detectors.^15^^,^^16^

In this work, we present a simple light emitting diode (LED)-based wearable multi-distance CW SWIR probe for the quantification of water and lipid content in tissue. We also describe the implementation of a deep neural network (DNN) for the mapping of diffuse reflectance measurements to water and lipid estimates. While DNN inverse models are becoming increasingly common, they are still infrequently utilized in the diffuse optics field, especially with SWIR technology. In the following sections, we first detail the design considerations, including a comparison of chromophore recovery performance between NIR and SWIR wavelengths in simulation. We then describe the physical layout of the probe, its control system, and its performance metrics. Finally, we validate its ability to quantify water and lipid *in vitro* through two different optical phantom experiments involving water and lipid emulsions.

## 2 Comparison of SWIR and NIR in Simulation

### 2.1 Wavelength and Source-Detector Separation Selection for Simulation

**Fig. 1:**
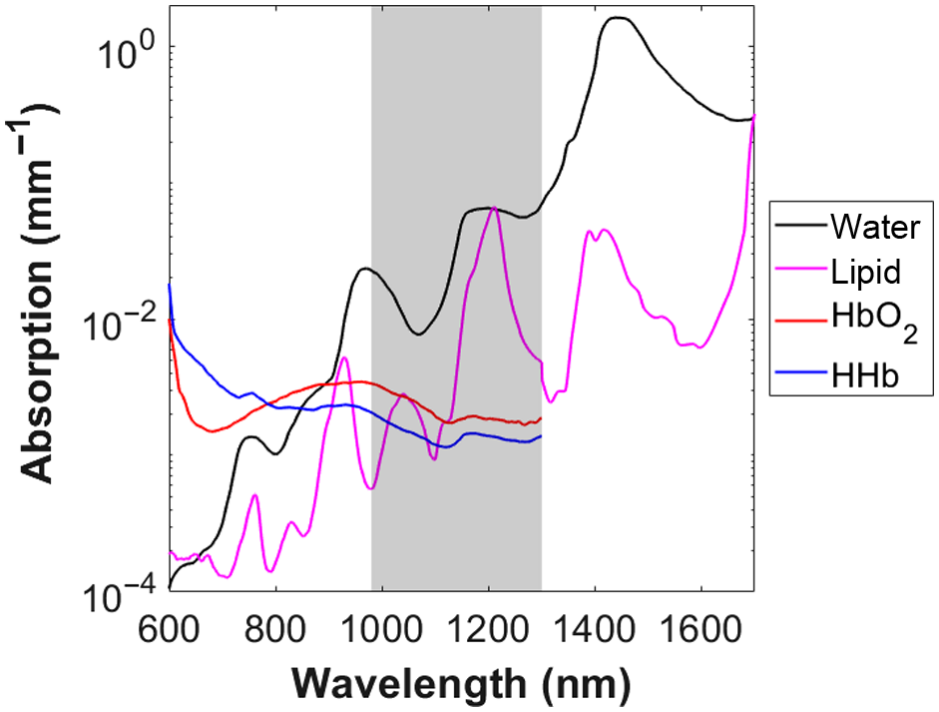
Absorption of endogenous chromophores in healthy breast tissue from 600 to 1700 nm (for water and lipid) and 600 to 1300 nm (for ${\mathrm{HbO}}_{2}$ and HHb). Tissue concentrations for this example are as follows: water = 51.5%, lipid = 40%, ${\mathrm{HbO}}_{2}=11.3\;\mu M$, HHb=5.3  μM. The shaded region (980 to 1300 nm) refers to desired SWIR wavelength region for our probe design.

Figure 1 shows representative absorption spectra of endogenous chromophores in tissue to provide a visual comparison of the NIR and SWIR wavelength bands. Absorption spectra were computed using published extinction spectra.^17^^–^^19^ Tissue concentrations of water, oxy-hemoglobin (${\mathrm{HbO}}_{2}$), and deoxy-hemoglobin (HHb) are average values reported for healthy breast tissue, with lipid estimated at 40% by volume.^20^ As seen in this figure, water absorption overtakes that of hemoglobin past ∼900  nm, whereas lipid exceeds hemoglobin absorption past ∼1100  nm for this tissue type. Water absorption becomes very high past 1400 nm, limiting imaging penetration depth and signal levels. A SWIR window of 980 to 1300 nm (shaded region) in Fig. 1) was chosen for the probe illumination based on these considerations. Discrete wavelengths at 980, 1200, and 1300 nm were then chosen based on LED availability. Water absorption is relatively high at all three of these wavelengths, whereas lipid absorption has a peak near 1200 nm. For the NIR, 900, 930, and 970 nm were chosen based on spectral features commonly exploited and LED availability. Four source-detector (S-D) separations (7, 10, 13, and 16 mm) were included in the simulations. In total, this resulted in 12 unique wavelength/separation pairs for the SWIR and NIR.

### 2.2 Simulation Workflow

**Fig. 2:**
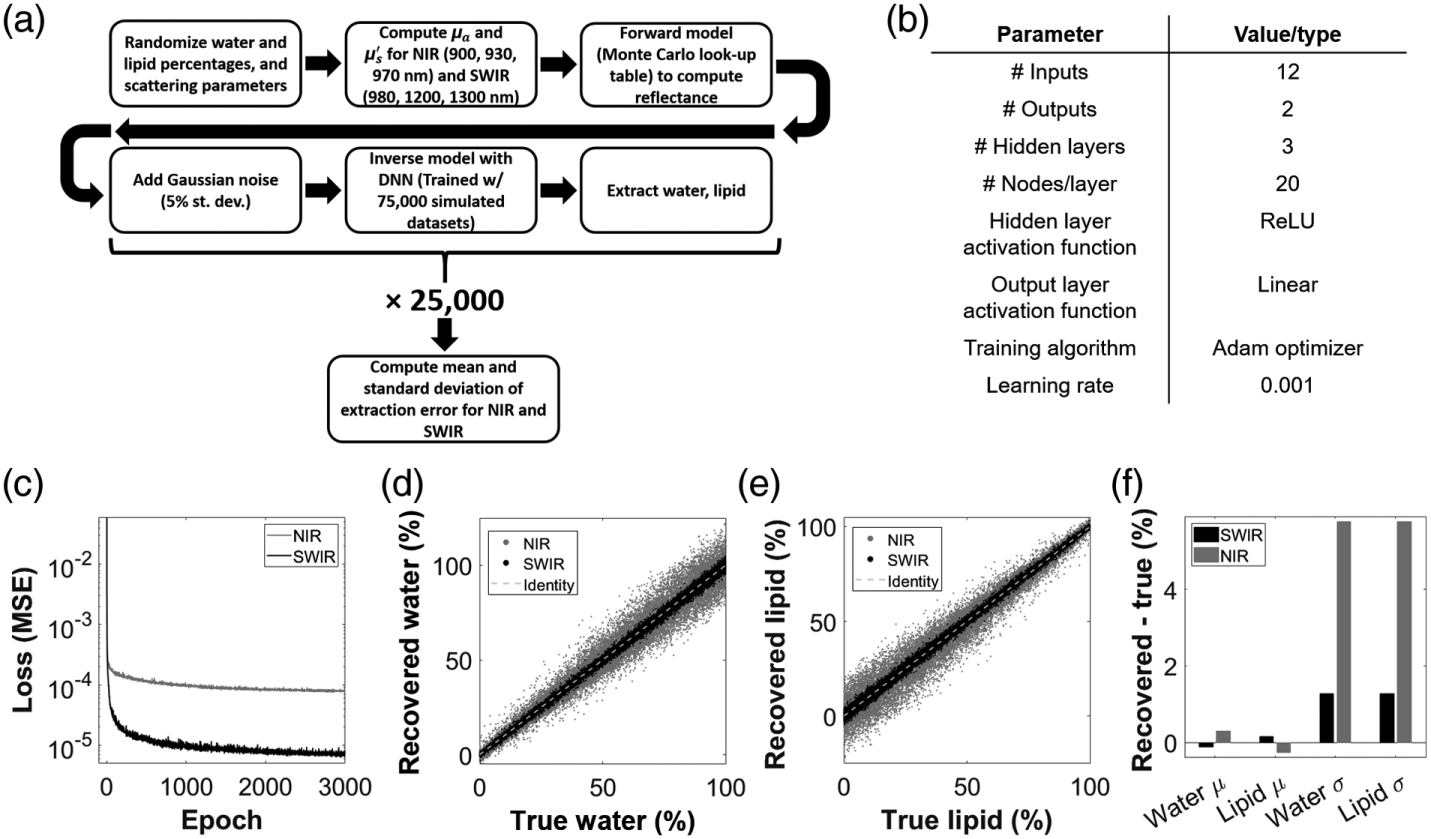
(a) Simulation flowchart for the comparison of SWIR and NIR wavelengths. (b) A table of architecture and training parameters for the DNNs used in this study. (c) Loss (MSE) as a function of epoch during training for both the SWIR and NIR DNNs. (d) All 25,000 recovered water values plotted versus ground truth for SWIR and NIR wavelengths. (e) Same as (d), but for lipid. (f) Bar plots of the mean and standard deviation of differences between recovered and ground truth values for both SWIR and NIR.

Simulations were performed using both Matlab and Python, and the workflow is described here and summarized in Fig. 2(a). For a single simulation, water, lipid, and scattering properties were randomized for the simulated “sample.” For each simulation performed, water and lipid concentrations were randomly selected from uniform distributions between 0% and 100% and always summed to 100% (entire simulated sample comprised of just water and lipid). The percentage refers to percent by volume. A power law [Eq. (1)] described the scattering relationship with wavelength, with scattering amplitude (A) and slope (b) as the randomized properties

$$\mu_{s}^{\prime }(\lambda )=A*{\left(\frac{\lambda }{980\;\mathrm{nm}}\right)}^{-b}.$$ (1)

Possible amplitude values were defined as a uniform distribution from 0.2 to $10\;{\mathrm{mm}}^{-1}$ (referenced to 980 nm). Scattering slope values were defined as a normal distribution with μ=1.29, σ=0.52, which are typical slope values for soft tissue.^21^ Next, the absorption coefficient ($\mu_{a}$) and reduced scattering coefficient ($\mu_{s}^{\prime }$) were computed for each of the three SWIR and NIR wavelengths. Beer’s Law was used to compute $\mu_{a}$ (using the same extinction spectra from Sec. 2.1), and Eq. (1) was used to compute $\mu_{s}^{\prime }$. A pre-generated look-up table (LUT) of Monte Carlo-derived diffuse reflectance ($R_{d}$) values then mapped optical property pairs to $R_{d}$ values for each of the 12 unique wavelength/separation pairs, for both the SWIR and NIR groups (Monte Carlo simulations described in more detail in the following section). Zero-mean Gaussian noise was then added to the $R_{d}$ values, with σ=5%. These noise-added $R_{d}$ values were then input to an inverse model to recover water and lipid estimates. A fully connected DNN was used as the inverse model. This process was repeated 25,000 times, resulting in 25,000 sets of recovered water and lipid values for both the SWIR and NIR groups. These recovered values were compared to the ground truth values to compute the mean and standard deviation of extraction errors.

### 2.3 Forward and Inverse Models: Monte Carlo and DNN

White Monte Carlo simulations were run and the results were used to map optical property pairs to surface diffuse reflectance. Simulations were run in Matlab using Monte Carlo eXtreme.^22^ The sample geometry was defined as a slab with dimensions of 6  cm×6  cm×10  cm, approximating a semi-infinite medium. Optical properties were homogenous, with $\mu_{a}=0\;{\mathrm{mm}}^{-1}$ and $\mu_{s}^{\prime }$ ranging from 0.2 to $10\;{\mathrm{mm}}^{-1}$. Detectors were placed 7, 10, 13, and 16 mm from the source, and a steady-state illumination source fiber was used. Collected photons for each $\mu_{s}^{\prime }$ value were then scaled for a range of absorption values using the Beer-Lambert law, with $\mu_{a}$ ranging from 0.001 to $0.2\;{\mathrm{mm}}^{-1}$. Anisotropy factor g and index of refraction n were set to 0.7 and 1.435, respectively, which are values for lipid-water emulsions adapted from Flock et al.^23^ and Aernouts et al.^24^ After the Monte Carlo simulations were completed, the $\mu_{a}$, $\mu_{s}^{\prime }$, and resultant $R_{d}$ values at each S-D separation were saved in a LUT.

The DNN was implemented in Python using the Keras library. The architecture and training parameters are summarized in Fig. 2(b) and are briefly described here. The DNN consists of an input layer with 12 nodes ($R_{d}$ for each wavelength/separation pair), 3 hidden layers, each with 20 nodes, and an output layer with 2 nodes (recovered water and lipid). The rectified linear unit was selected as the activation function for hidden layer nodes, and a linear function was the activation function for the output layer. The Adam optimizer was the training algorithm with a learning rate = 0.001. The loss function (or error function) was the mean squared error (MSE) of water and lipid. A trial-and-error approach was used to select the number of hidden layers and nodes/layer, initially exploring 1 to 6 hidden layers and 10 to 100 nodes/layer. It was found that adding more layers beyond 3 had diminishing returns in error minimization, and likewise beyond 20 nodes/layer. However, this optimization was coarse, and other functional architectures exist. The chosen activation function, training algorithm, learning rate, and loss function are all commonly used for fully connected DNNs. For training data, 75,000 sets of water, lipid, and $R_{d}$ values (generated using the same Monte Carlo LUTs described previously) were constructed in Matlab and imported into Python, using the same randomization process and parameters described for the 25,000 test datasets in Sec. 2.2. These parameters are also reported in Table S1 in the Supplementary Material. The $R_{d}$ inputs were log-normalized prior to training. The spectral constraints described in Sec. 2.2, namely (1) absorption limited to a linear sum of water and lipid contributions and (2) the wavelength dependence of scattering defined by a power law, provided important constraints during training, which helped to reduce the solution space. Two DNNs were trained, one for SWIR and one for NIR. Initially, automatic stopping was implemented during training, stopping when the mean loss over the previous 200 epochs was no longer decreasing. This occurred after ∼2000 epochs for both SWIR and NIR. To be cautious, the final DNNs used for comparison were trained with 3000 epochs. All subsequent DNNs in this study (after the SWIR versus NIR comparison), however, were trained with 2000 epochs.

### 2.4 Simulation Results

Figure 2(c) illustrates the first indication that SWIR outperforms NIR in water and lipid recovery by showing the MSE during training of the two DNNs. The NIR MSE plateaued at around $10^{-4}$, whereas the SWIR MSE dropped below $10^{-5}$, indicating that, even prior to noise addition, the SWIR wavelengths were able to achieve a lower error in water and lipid extractions. Figures 2(d) and 2(e) show plots of recovered water and recovered lipid versus their ground truths, respectively, for the 25,000 noise-added test sets described in Sec. 2.2. It is evident that for both water and lipid, the spread of recovered values more closely approaches the identity line for the SWIR wavelengths. This is especially true for high water/low lipid samples, with more comparable errors for near-100% lipid samples. Figure 2(f) quantifies this error, showing the mean and standard deviation of the difference between recovered and true values for all 25,000 sets of water and lipid values. For SWIR wavelengths, the errors for water and lipid recovery were −0.1%±1.3% and 0.2%±1.3%, respectively. The NIR error was higher, with values for water and lipid of 0.3%±5.8% and −0.3%±5.8%, respectively. These results suggest that the chosen SWIR wavelengths outperform NIR wavelengths in water and lipid recovery.

## 3 Instrument Design and Characterization

### 3.1 Printed Circuit Board and Probe Housing

The system consists of two components: a probe and a microcontroller (FRDM-K64F). The probe has one InGaAs PIN photodiode (G12180-30, Hamamatsu), four 980 nm LEDs (MTE9730CP), four 1200 nm LEDs (MTSM0012-843-IR), and four 1300 nm LEDs (MTSM0013-199-IR). All LEDs were purchased from Marktech. The photodiode has an active area of $∼7\;{\mathrm{mm}}^{2}$ and is sensitive to the 900 to 1700 nm wavelength range. The quantum efficiency at the illumination wavelengths is as follows: ∼63% at 980 nm, ∼83% at 1200 nm, ∼86% at 1300 nm. LEDs were positioned at four different distances from the photodiode: 7, 10, 13, and 16 mm. These were the minimal distances achievable given the size of the optical elements. A transimpedance amplifier (OPA3S32), with three integrated switchable gain levels (0.5, 1, and 10 MΩ), was used to amplify and convert the photodiode current into voltage. This output voltage is read by a 16-bit analog to digital converter embedded on the microcontroller. The intensity of the twelve LEDs is controlled with the 12-bit digital to analog converter (DAC) embedded on the microcontroller. The DAC output voltage is translated into current with an operational amplifier (TLV272IS-13) and NPN bipolar junction transistor (TTC1949-GR,LF). The microcontroller communicates with the probe through a high-definition multimedia interface (HDMI) connection, and the user can interact with the system with a Python GUI (graphical user interface). The sampling rate of the system was set to 1 Hz.

**Fig. 3:**
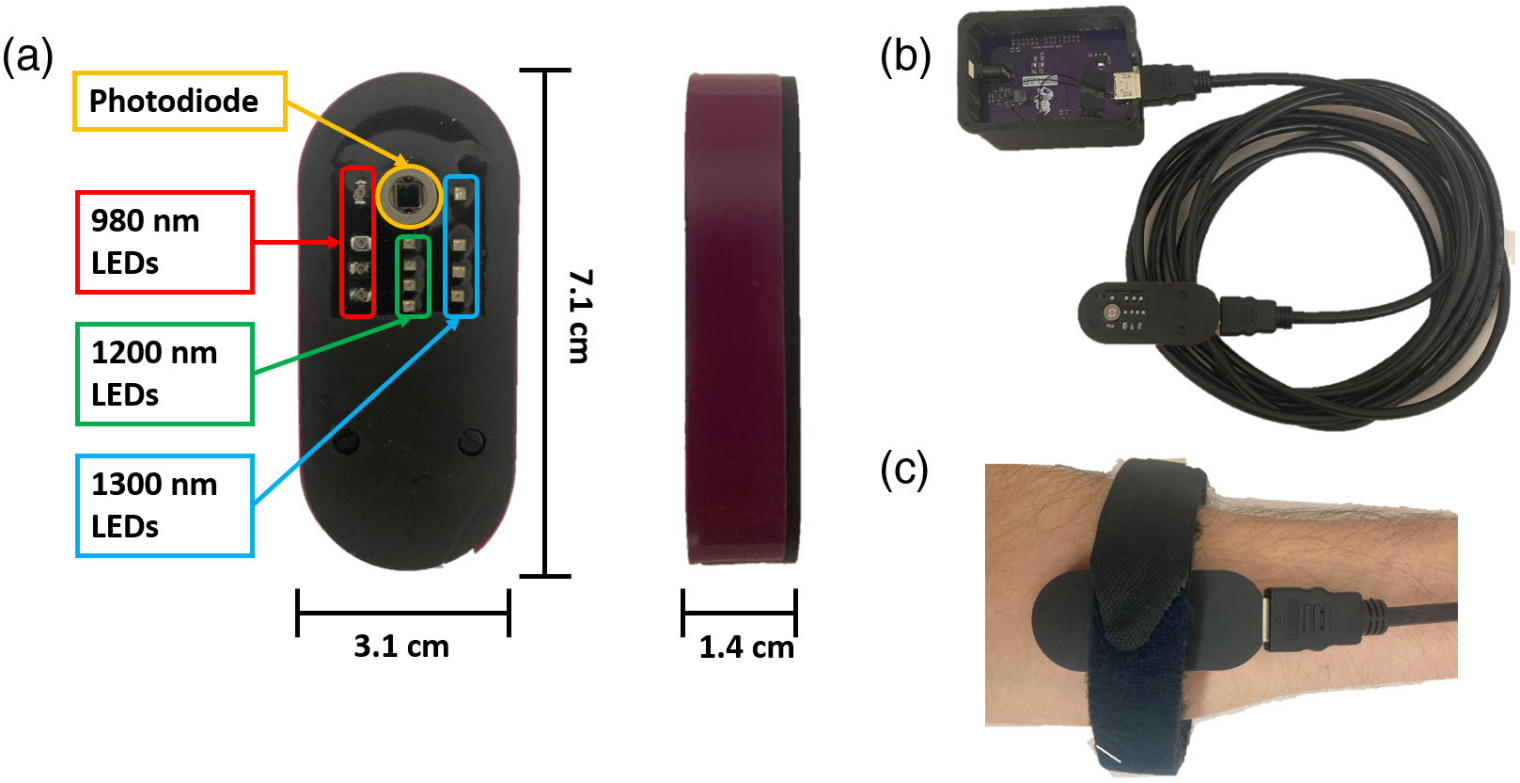
(a) Sample-facing and side views of the encased SWIR probe, with optical elements labeled. (b) The probe attached to its external control hardware (the microcontroller) via HDMI. (c) An example of how the probe might be worn on the forearm of a human subject with a Velcro strap.

The SWIR probe is shown in Fig. 3(a) and the probe with control hardware is shown in Fig. 3(b). Figure 3(c) shows the probe adhered to a forearm with Velcro. The probe was encased in a custom 3D-printed plastic housing with overall dimensions of 7.1  cm×3.1  cm×1.4  cm. Black silicone was used to isolate the optical elements. The silicone (Ecoflex-030, Smooth-On Inc.) and black dye (Silc-Pig, Smooth-On Inc.) are skin-safe and were mixed together with a curing agent, de-gassed in a vacuum chamber for 15 min, and poured directly onto the exposed PCB (printed circuit board) surface.

### 3.2 Performance Characterization

Signal-to-noise ratio (SNR) and system drift were measured on a liquid optical phantom containing 1% Intralipid solution in water. Both the water and lipid absorb light at SWIR wavelengths, and the lipid micelles are highly scattering, creating a diffusive medium. The optical properties of 1% Intralipid across the SWIR probe wavelengths, calculated using Beer’s Law and Mie theory, range from 0.04 to $0.13\;{\mathrm{mm}}^{-1}$ for $\mu_{a}$ and 0.49 to $0.78\;{\mathrm{mm}}^{-1}$ for $\mu_{s}^{\prime }$. The probe was wrapped tightly in clear plastic wrap and fixed in a slightly submerged position within the liquid phantom during measurement acquisition.

For the SNR measurement, the signal for each S-D pair was recorded for 1 min at the fixed rate of 1 Hz. An initial background measurement was recorded with all of the LEDs off prior to the acquisition. SNR was then computed as

$$10\cdot \log_{10}(\frac{\text{mean}(V_{p}-V_{d})}{\mathrm{std}(V_{p}-V_{d})}),$$ (2)

where $V_{p}-V_{d}$ is the difference between each sample voltage and the background dark voltage. This dark subtraction was performed for all subsequent measurements. The SNRs for S-D separations ≤10  mm are >30  dB (Table 1).

**Table 1:** Performance characteristics of SWIR probe. SNR and drift values are shown for just the shortest and longest S-D separations (7 and 16 mm).

Parameter	Value (S-D separation)
Sampling rate	1 Hz
SNR	
980 nm	36 dB (7 mm)
	22 dB (16 mm)
1200 nm	42 dB (7 mm)
	32 dB (16 mm)
1300 nm	41 dB (7 mm)
	21 dB (16 mm)
Drift	
980 nm	≤1% V/h (7 mm)
	≤6% V/h (16 mm)
1200 nm	≤1% V/h (7 mm)
	≤4% V/h (16 mm)
1300 nm	≤1% V/h (7 mm)
	≤19% V/h (16 mm)

For the drift measurement, the signal for each S-D pair was recorded for 1 h. The voltage and time data were then fit to a linear function to quantify the slope, which was normalized to the initial voltage and multiplied by 100 to get units of % V/h. Drift values for S-D separations ≤10  mm were <1.1% V/h (Table 1).

## 4 *In Vitro* Validation

### 4.1 Water-in-Oil Emulsion Phantoms

Five water-in-oil emulsion phantoms were fabricated using soybean oil, water, and Triton X-100 as the emulsifier as described in Merritt et al.^7^ Lipid concentrations were 65% to 85%, in steps of 5%. High (≥65%) lipid concentrations were used to minimize scattering differences across phantoms, which occurs as a result of dependent scattering.^24^ The SWIR probe was wrapped in clear plastic wrap and submerged in the emulsion phantoms. The probe optical elements were at least 1.5 cm from the phantom edge. Ten seconds of data were acquired at 1 Hz for each phantom measurement.

**Fig. 4:**
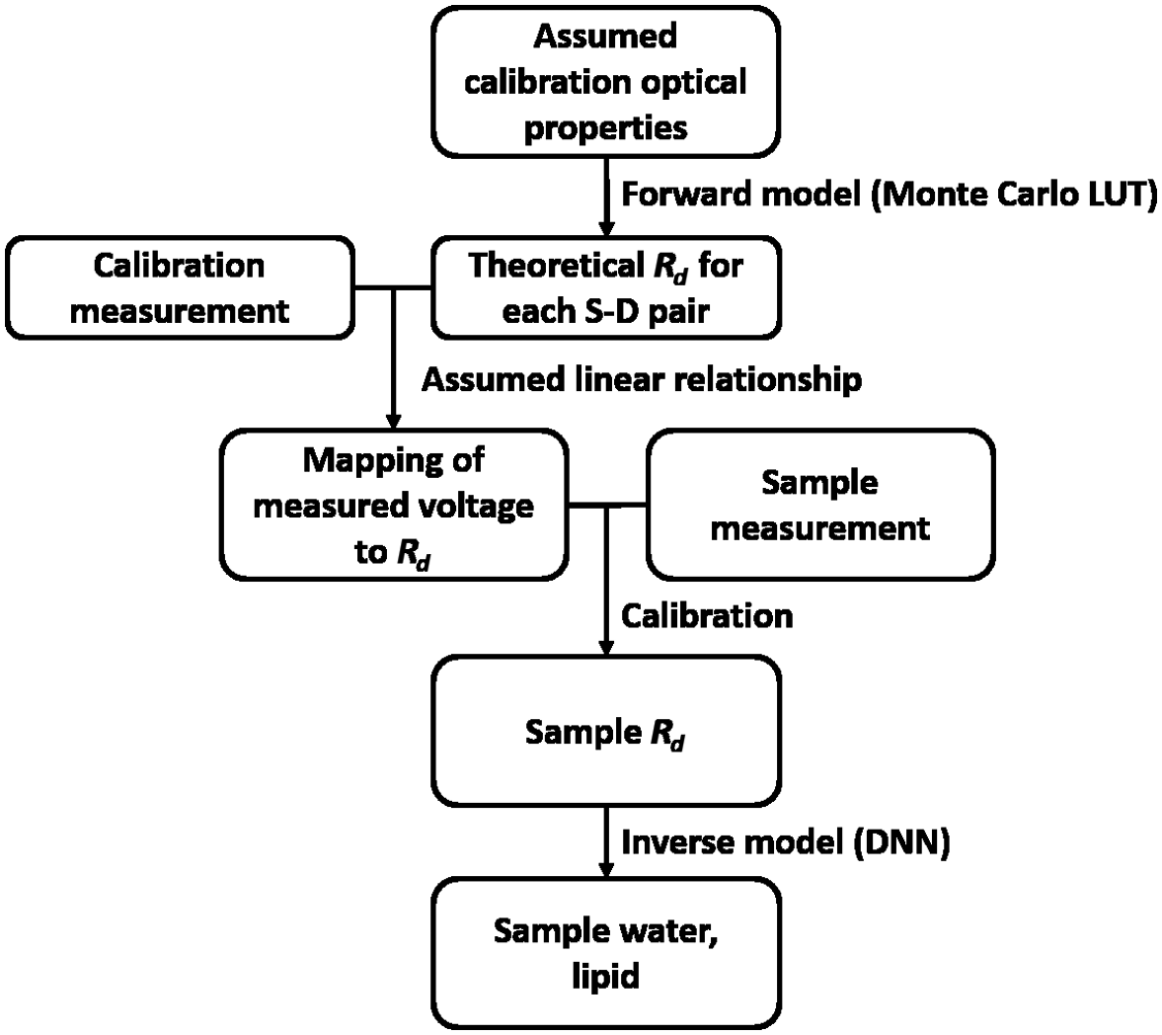
Data processing flowchart for *in vitro* validation experiments.

The processing pipeline is depicted in Fig. 4. Measured sample and dark voltage measurements were first gain-corrected, and all sample voltage measurements were dark subtracted. Next, a forward model (Monte Carlo LUT) was used to map the known optical properties ($\mu_{a}$ and $\mu_{s}^{\prime }$) of the calibration phantom to a theoretical $R_{d}$ value. As the photodiode operates linearly with incident light power, the $R_{d}$ value was divided by the calibration measurement voltage to get a linear scaling factor, specific to each S-D pair. The corrected sample voltage was then multiplied by this scaling factor to compute $R_{d}$ for that S-D pair.

The 85% lipid phantom was used as the calibration phantom. Beer’s Law was used to compute $\mu_{a}$, and the empirical formula given by Aernouts et al.^24^ was used to compute $\mu_{s}^{\prime }$. This formula defines $\mu_{s}^{\prime }$ as a function of wavelength and lipid concentration for emulsions up to 20% lipid concentration. However, the dependency on concentration nearly flattens at this upper limit due to dependent scattering. Therefore, we approximated the scattering values for 85% lipid as being equal to those of 20% lipid. These optical properties ranged from 0.04 to $0.13\;{\mathrm{mm}}^{-1}$ for $\mu_{a}$ and 4.8 to $8.9\;{\mathrm{mm}}^{-1}$ for $\mu_{s}^{\prime }$ for the three measurement wavelengths. The DNN architecture was nearly identical to the DNN described in Sec. 2, except the 16 mm S-D separation measurements were removed due to low signal, resulting in 9 $R_{d}$ inputs to the DNN. The DNN was trained with 75,000 sets of 9 $R_{d}$ values. Additional information related to the training data is given in Table S1 in the Supplementary Material.

**Fig. 5:**
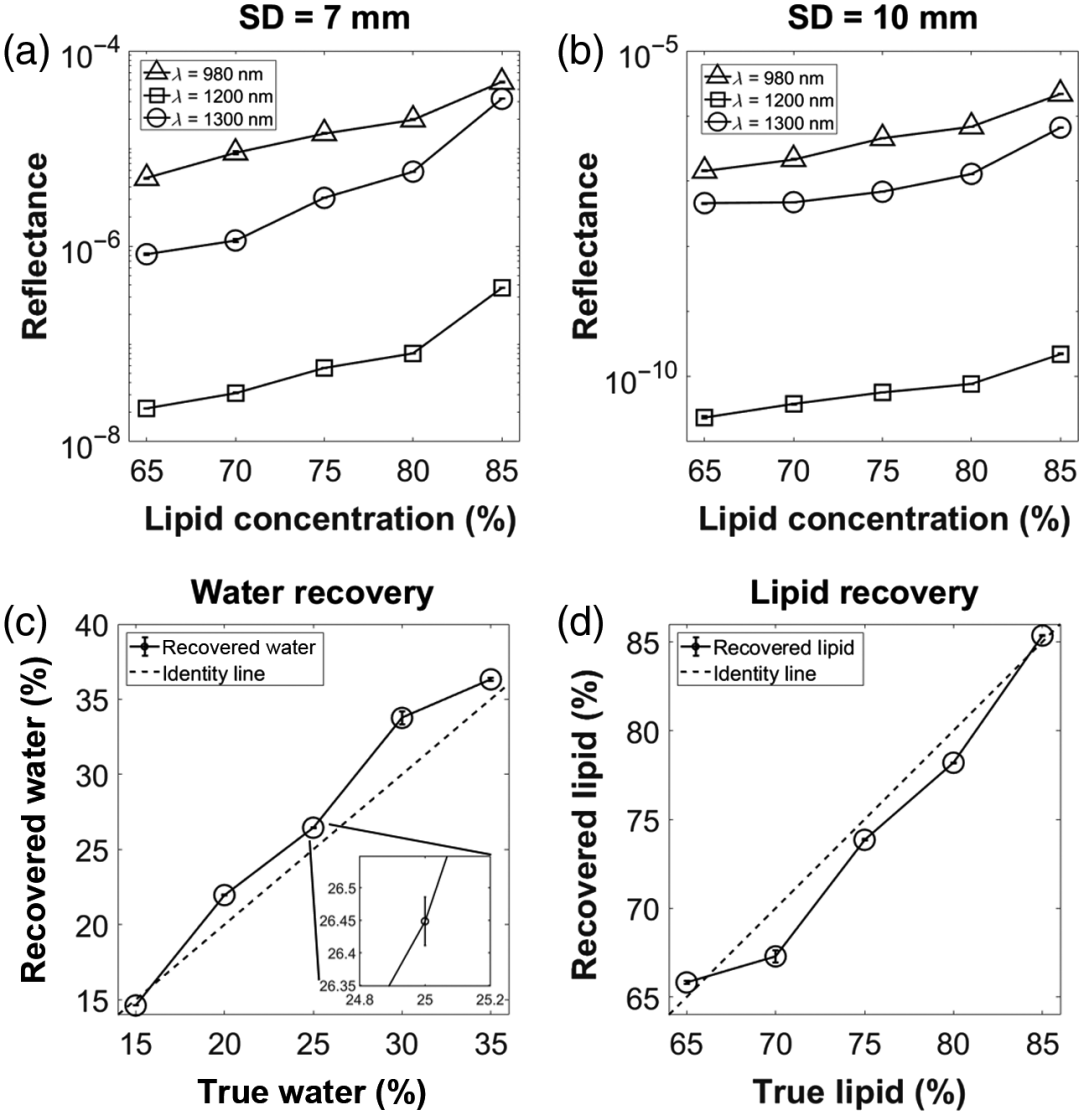
a) $R_{d}$ versus lipid concentration for S-D separation = 7 mm. (b) Same as (a), but for S-D separation = 10 mm. (c) Recovered water versus true water. The inset zooms in on the 25% water data point, showing the length of the error bars. These error bars refer to the standard deviation across the 10 consecutive time points that were acquired for each phantom. All four subfigures have these error bars, but they are barely visible when zoomed out. (d) Same as (c), but for lipid recovery.

Figures 5(a) and 5(b) show $R_{d}$ versus lipid concentration for the 7 and 10 mm separations. $R_{d}$ increased as lipid concentration increased and water concentration decreased. This is expected at 980 and 1300 nm as water has high absorption compared to lipid. While lipid’s extinction coefficient at 1200 nm is slightly higher than water’s, it is very close to an isosbestic point for the two chromophores, and the LED illumination is broad (∼80  nm full-width at half maximum). This likely explains why the 1200 nm data appears to behave similarly to the other wavelengths. Also, $R_{d}$ at 1200 nm is below that of both 980 and 1300 nm, which is consistent with the fact that both water and lipid have relatively high absorption here. Figures 5(c) and 5(d) show the correlations between recovered water and lipid, respectively, and their ground truth values. Both the water and lipid estimates follow the identity line closely. Removing the calibration phantom from the sample set, the mean ± standard deviation of the error (defined as error = estimated – true) was 2.1%±1.1% for water recovery and −1.2%±1.5% for lipid recovery. These results indicate that the SWIR probe is sensitive to variation in water and lipid content, and that, given certain *a priori* information, it can accurately quantify water and lipid content *in vitro*.

### 4.2 Dilution of Oil-in-Water Emulsion with $D_{2}O$

To further validate the SWIR probe’s sensitivity to water concentrations over a wide range, measurements were taken on a solution of 1% Intralipid in pure water ($H_{2}O$), which was serially diluted with 1% Intralipid in deuterium oxide ($D_{2}O$). $D_{2}O$ molecules consist of two $H^{2}$ isotopes instead of $H^{1}$, and their absorption is greatly reduced in the SWIR compared to $H_{2}O$ molecules.^25^ The initial 1% Intralipid solution was measured in a well embedded in a diffusive solid silicone phantom. The well had a volume of 150 ml and was 2.5 cm deep. The SWIR probe was wrapped in clear plastic wrap and fixed at the surface of the solution, slightly submerged. Data acquisition was started and measurements were taken for 10 s at 1 Hz. Ten phantoms were measured, with $H_{2}O$ concentrations ranging from 99% to 9% in steps of 10%. The concentration of scattering lipid particles was preserved across all phantoms.

The 1% Intralipid in $H_{2}O$ solution was used as the calibration phantom for this experiment, using the same known optical properties listed in Sec. 3.2. The Monte Carlo LUTs used to produce the DNN training data were generated with the index of refraction set to 1.33 to reflect the low lipid concentration. The combinations of $H_{2}O$ and lipid were not constrained to sum to 100%, as the decreasing $H_{2}O$ concentration was replaced by $D_{2}O$, not lipid. Furthermore, we assumed that $D_{2}O$ absorption was collinear with $H_{2}O$ absorption but with 1/10th the magnitude, an estimate based on the extinction spectra reported in Wang et al.^25^ This $D_{2}O$ $\mu_{a}$ contribution was taken into account when generating the training data. Finally, the scattering was fixed to be equal to that of 1% Intralipid. All 12 S-D pairs, spanning all four S-D separations, were input to this experiment’s DNN. The DNN training data details are listed in Table S1 in the Supplementary Material.

**Fig. 6:**
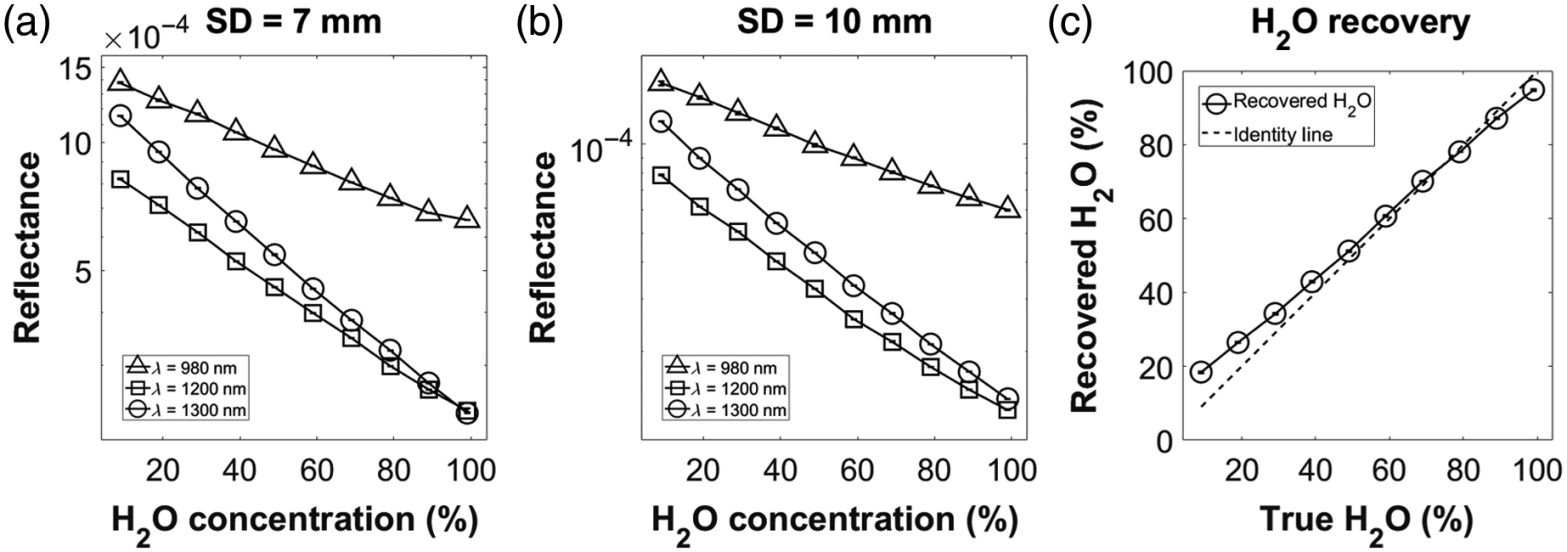
(a) $R_{d}$ versus H2O concentration for S-D separation = 7 mm. (b) Same as (a), but for S-D separation = 10 mm. (c) Recovered $H_{2}O$ versus ground truth compared to the identity line. For all subfigures, error bars are presented as the standard deviation across the 10 consecutive time points for each phantom measurement. Error bars are present but barely visible.

Figures 6(a) and 6(b) show $R_{d}$ as a function of $H_{2}O$ concentration for the 7 and 10 mm S-D separations respectively. The slope of $R_{d}$ is steepest for 1300 nm, explained by the relatively high absorption from $H_{2}O$ at this wavelength compared to the other two. We can assume that these trends in $R_{d}$ are strictly absorption-dependent since the scattering lipid particle concentration was held constant. Figure 6(c) shows the recovered $H_{2}O$ concentration compared to the ground truth concentration. The estimates are within 4% of the ground truth from 49% to 99% $H_{2}O$ and are within 10% for all data points. Over the full range of concentrations, the mean ± standard deviation of the error was 3.1%±3.7%. This suggests that the SWIR probe has sensitivity to water at all concentrations, although the accuracy of water estimation appears to degrade at very low concentrations.

## 5 Discussion

We have presented here a CW wearable SWIR probe with LEDs that span 3 wavelengths (980, 1200, 1300 nm) and 4 S-D separations (7, 10, 13, 16 mm), totaling 12 unique S-D pairs, and validated its ability to quantify water and lipid content *in vitro*. To our knowledge, this is the first wearable, LED-based SWIR probe capable of quantifying water and lipids.

There are a handful of prior reports showing that diffuse optical technologies are able to quantify *in vitro* water and lipid concentrations. Merritt et al.^7^ used a combined laser-based FD + broadband CW system with wavelengths from 650 to 1000 nm to estimate water and lipid concentrations of the same type of emulsion phantoms measured here. They included 12 phantoms in their study with water and lipid concentrations ranging from 35% to 94% and 6% to 63%, respectively. They reported water estimates within 2% of ground truth across this range and lipid estimates within 9%. This error manifested as a constant offset, and lipid recovery was highly linear. Lam et al.^5^ used a broadband CW system from 900 to 1000 nm to measure 5 similar emulsion phantoms with water and lipid concentrations ranging from 20% to 60% and 40% to 80%, respectively. Errors ranged from 1.1% to 8.4%, with a mean error of 3.7%±3.0%. Nachabe et al.^14^ achieved water and lipid errors of <5% over a wide variety of emulsion phantom types using a broadband CW system from 900 to 1600 nm. In that study, both custom and commercial (butter and margarine) phantoms were measured, including sonication of custom emulsion phantoms to vary scattering while holding water and lipid concentration constant. This was the only other demonstration of CW diffuse optical measurements beyond 1000 nm of such phantoms besides ours. Our emulsion phantom results, with errors of 2.1%±1.1% for water recovery and −1.2%±1.5% for lipid recovery, as well as 3.1%±3.7% in water recovery for the $D_{2}O$ dilution experiment, compare well to these previous studies. Furthermore, the three other studies used broadband light sources allowing for the acquisition of hyperspectral data, whereas our SWIR probe minimizes the instrument design using just three wavelengths.

The use of $D_{2}O$, which has been exploited previously for quantifying water absorption properties in the SWIR region,^26^ offers a unique advantage over other emulsion phantom experiments. Most prior studies have utilized emulsions in which both water and lipid concentrations were changed simultaneously. This strategy is limited in one sense because the changes in lipid micelle concentrations also change optical scattering, making it is difficult to know whether observed water sensitivity is in part conflated with scattering sensitivity, especially when lipid concentration is <20%. Pilvar et al.^16^ demonstrated this by showing how a small increase from 1.5% to 2.5% intralipid in water had a negligible effect on $\mu_{a}$, but a significant effect on $\mu_{s}^{\prime }$. This makes it challenging to predict how performance would translate to applications in which only water concentration varies while scattering stays constant or changes in an unknown direction, which may occur in tissue. The $D_{2}O$ experiment allowed for the confirmation of good sensitivity to water concentration changes over nearly the entire range of possible concentrations.

In this work, we utilized DNNs trained with Monte Carlo simulated data as our inversion model. Other common inversion models, such as an iterative analytical solution based on the P1 approximation to the radiative transport equation, were not applicable here due to the relatively high absorption and low scattering at SWIR wavelengths. Others have utilized LUT-based inversion models that directly map $R_{d}$ to $\mu_{a}$ and $\mu_{s}^{\prime }$ on a per wavelength basis, but the addition of spectral constraints requires an iterative error minimization process, which is especially complex and computationally costly for highly discretized LUTs.^27^^–^^29^ A DNN, in contrast, enables inverse solution to be found non-iteratively and rapidly. DNN inversion models have been utilized for a number of different diffuse optical modalities,^30^ including in our prior work.^31^^,^^32^ One important aspect of the DNN inversion used here was the incorporation of spectral constraints on the training data. While the DNN was not spectrally constrained per se, the constraint was effectively imposed on the inversion process through the training process.

Limitations of this study include the fact that constraints were applied to the training data in specific cases. For example, for the emulsion phantom study, it was assumed that water and lipid concentrations summed to 100%. In tissue samples, other chromophores, such as hemoglobin, may be present in abundance, so this constraint would have to be removed. Our $D_{2}O$ dilution experiment involved training a DNN without this constraint, but only one other chromophore (lipid) was present, and it was held constant. It remains to be seen if this method would be robust enough to quantify simultaneously varying amounts of both water and lipid in the presence of other chromophores. The DNN used for the $D_{2}O$ experiment was further constrained as the scattering properties of the training data were fixed to that of 1% Intralipid solution in water. For future *in vivo* measurements, a scattering estimate could be assumed for different tissue types. That said, the water-in-oil emulsion phantom study did not incorporate a fixed scattering constraint, which suggests that the spectral constraints with our method may be sufficient for recovering water and lipid with unknown scattering in other applications.

In this work, we first confirmed the theoretical performance benefits of SWIR over NIR wavelengths in simulation, designed and fabricated a novel wearable SWIR probe, and characterized its performance. We then validated its sensitivity to water and lipid, and in doing so demonstrated its ability to quantify concentrations of these chromophores accurately *in vitro*. This opened the door to human studies that aim to test the functionality and utility of this probe *in vivo* for a variety of applications.

## Code, Data, and Materials Availability

Scripts and associated instructions for performing each step of the SWIR versus NIR comparison in simulation are provided in a repository on Github: https://github.com/BU-BOTLab/SWIRprobe_paper_JBO2023. Furthermore, the exact DNNs used for this comparison are located in the same repository. All subsequent DNNs are adapted from the scripts provided there, with further information available upon request.
