# Supplementary material for: Shortwave infrared diffuse optical wearable probe for quantification of water and lipid content in emulsion phantoms using deep learning
Source: J Biomed Opt. 2023 Jun 12;28(9):094808. doi: 10.1117/1.JBO.28.9.094808 (PMC10258729; doi:10.1117/1.JBO.28.9.094808)
Supplement: Supplementary file 1 [file JBO_028_094808_SD001.pdf]

Table S1 Parameters that describe the training data used for each of the DNNs in this study.

Training data/DNN for:	g	n	Scattering amplitude (mm^{-1})	Scattering slope	Water + Lipid = 1?	SD separations (mm)
SWIR vs. NIR comparison	0.7	1.435	Uniform distribution between 0.2 and 10	Normal distribution (μ = 1.29, σ = 0.52)	Yes	7, 10, 13, 16
Emulsion phantom experiment	0.7	1.435	Uniform distribution between 0.2 and 10	Normal distribution (μ = 1.29, σ = 0.52)	Yes	7, 10, 13
D_2O experiment	0.7	1.33	0.78	1.4	No	7, 10, 13, 16
